# Supplementary material for: Development of a computer-aided design software for the quantitative evaluation of aesthetic damage
Source: PLoS One. 2019 Dec 18;14(12):e0226322. doi: 10.1371/journal.pone.0226322 (PMC6919621; doi:10.1371/journal.pone.0226322)
Supplement: S1 Code site — 1. (ZIP) [file pone.0226322.s001.zip › [EESC_jr][Projeto]Relatorio_07_21/reports/templates_zip/gentelella-master/vendors/Flot/examples/ajax/index.html]

Flot Examples: AJAX


## AJAX

Example of loading data dynamically with AJAX. Percentage change in GDP (source: Eurostat). Click the buttons below:

The data is fetched over HTTP, in this case directly from text files. Usually the URL would point to some web server handler (e.g. a PHP page or Java/.NET/Python/Ruby on Rails handler) that extracts it from a database and serializes it to JSON.

First dataset
[ see data ]

Second dataset
[ see data ]

Third dataset
[ see data ]

If you combine AJAX with setTimeout, you can poll the server for new data.

Poll for data

Copyright © 2007 - 2014 IOLA and Ole Laursen
